# Genetic assignment of fisheries bycatch reveals disproportionate mortality among Alaska Northern Fulmar breeding colonies

**DOI:** 10.1111/eva.13357

**Published:** 2022-03-04

**Authors:** Diana S. Baetscher, Jessie Beck, Eric C. Anderson, Kristen Ruegg, Andrew M. Ramey, Scott Hatch, Hannah Nevins, Shannon M. Fitzgerald, John Carlos Garza

**Affiliations:** ^1^ 8787 University of California Santa Cruz Santa Cruz California USA; ^2^ NOAA Southwest Fisheries Science Center Santa Cruz California USA; ^3^ Oikonos Ecosystem Knowledge Santa Cruz California USA; ^4^ 3447 Colorado State University Fort Collins Colorado USA; ^5^ U.S. Geological Survey Alaska Science Center Anchorage Alaska USA; ^6^ Institute for Seabird Research and Conservation Anchorage Alaska USA; ^7^ NOAA Alaska Fisheries Science Center Seattle Washington USA; ^8^ Present address: Auke Bay Laboratories NOAA Alaska Fisheries Science Center Juneau Alaska USA

**Keywords:** bycatch, genetic stock identification, microhaplotype, Northern Fulmar, population structure, utilization distribution

## Abstract

Global fisheries kill millions of seabirds annually through bycatch, but little is known about population‐level impacts, particularly in species that form metapopulations. U.S. North Pacific groundfish fisheries catch thousands of Northern Fulmars (*Fulmarus glacialis rodgersii*) each year, making fulmars the most frequently caught seabird in federally managed U.S. fisheries. Here, we used genetic stock identification to assign 1,536 fulmars sampled as bycatch to one of four Alaska breeding colonies and quantified the similarity of bycatch locations at sea among colonies. We found disproportionately high bycatch from the Pribilof Islands (6% of metapopulation, 23% of bycatch), and disproportionately low bycatch from Chagulak Island (34% of metapopulation, 14% of bycatch). Overlap between fisheries and colony‐specific foraging areas diverge more during the summer breeding season, leading to greater differences in bycatch susceptibility. Contemporary and historical gene flow likely contributes to low genetic differentiation among colonies (F_ST_ = 0.003–0.01), yet these values may not represent present connectivity. Our findings illustrate how genetic stock identification can link at‐sea threats to colonies and inform management to reduce bycatch from impacted colonies.

## 1 INTRODUCTION

Anthropogenic impacts on wide‐ranging vertebrates can be challenging to quantify due to broad geographic distributions and exposure to threats during seasonal use of multiple habitats. One such impact is fisheries bycatch, which kills millions of seabirds globally each year through incidental capture (Dias et al., 2019). Although bycatch can be reduced by mitigation techniques (Melvin et al., 2019), entanglement in fishing gear remains one of the greatest conservation threats to seabirds (Anderson et al., 2011; Dias et al., 2019; Lewison et al., 2014), whose populations are vulnerable due to their long‐lived, low‐fecundity life histories (Tuck et al., 2015). While negative effects of bycatch are documented for well‐monitored species (e.g., Pardo et al., 2017), impacts to less closely monitored species with dispersed metapopulations are uncertain.

Reducing seabird bycatch requires understanding the patterns of spatial and temporal overlap between fisheries and birds that lead to mortality (Lewison et al., 2014). For seabirds, these patterns are largely determined by foraging behavior, and the impacts of bycatch potentially exacerbated when seabirds exist as metapopulations (i.e., local populations that interact by individuals moving among populations; Hanski & Gilpin, 1991), such as species with colonies spread across islands (Inchausti & Weimerskirch, 2002). Many seabirds exhibit evidence of restricted gene flow and population differentiation among breeding colonies (Friesen, 2015). Thus, disproportionate bycatch from distinct populations can have genetic (Edwards et al., 2001), population‐level (Inchausti & Weimerskirch, 2002), and ecological (e.g., Thoresen et al., 2017) repercussions to the larger metapopulation. Additionally, climate change, especially at high latitudes, can intensify anthropogenic impacts to seabirds by shifting the timing and intensity of primary production, altering foraging habitat or the distribution of prey, and restricting or otherwise changing species’ distributions (e.g., Yati et al., 2020).

The North Pacific groundfish and Pacific halibut fisheries (hereafter “groundfish fisheries”) in the Alaska region make up more than half of the total seabird bycatch in federally managed U.S. fisheries (Benaka et al., 2019). These fisheries extract the largest volume of catch of all U.S. fishery regions (NMFS, 2021) from one of the most productive marine regions (Marshak & Link, 2021). Groundfish fisheries operate in the Bering Sea, Aleutian Islands, and Gulf of Alaska. In each region, oceanographic processes act to concentrate nutrients, contributing to high biomass production of benthic commercial fish species. In the Bering Sea, nutrient‐rich waters flowing through the Bering Strait are derived from spring phytoplankton blooms that sink and are concentrated by thermal stratification of surface and subsurface waters, and the thermal barrier of the Bering Sea cold pool (Grebmeier et al., 2015). In the Gulf of Alaska, primary production is fueled by the Alaska Current, which receives substantial freshwater input and terrestrial nutrients from weathering. These waters eventually move north and west along the Gulf of Alaska coast, becoming the Alaska Stream and flowing along the southern boundary of the Aleutian Islands where they support benthic ecosystems and groundfish stocks (Stabeno et al., 2004).

The Northern Fulmar (*Fulmarus glacialis rodgersii*; hereafter “fulmar”), a Procellarid seabird, comprises more than half of the seabird bycatch in groundfish fisheries, making it the most incidentally caught seabird species in the United States (Benaka et al., 2019), and one of the most frequently caught seabird species in longline fisheries worldwide (Anderson et al., 2011). Although groundfish fisheries reduced fulmar bycatch by an order of magnitude in 2002 with the use of bird scaring lines, an estimated 4701–4440 fulmars were caught per year from 2007 to 2017 (Eich et al., 2018).

Fulmars are long‐lived, and like other Procellarid seabirds, they mature slowly, and produce one chick per year. Typically, fulmars start breeding around 10 years of age (8 years for males, 12 years for females; Dunnet, 1992). Throughout the year, they travel long distances (1000s of km) to scavenge prey on the surface of pelagic waters, but during the breeding season (May to September) adults become central‐place foragers as both sexes provide parental care (Mallory et al., 2020). During this time, fulmars are typically within 500 km of their breeding colony, and distance from colony is a key factor that explains fulmar at‐sea distributions in the Bering Sea (Renner et al., 2013). This same study also identified oceanographic (bathymetry and sea surface temperature) and fishery‐related variables strongly correlated with fulmar distributions, indicating that the availability of fisheries discards influences foraging behavior in this region (Renner et al., 2013).

The fulmar population in the Alaska region is estimated at 1.5 million individuals (Hatch, 1993), 99% of which breed at four colonies in the Bering Sea, Gulf of Alaska, and Aleutian Islands. Population estimates place 440,000–500,000 fulmars at each of the three colonies (Chagulak Island, St. Matthew and Hall Islands, and the Semidi Islands) and just under 80,000 birds at the Pribilof Islands (St. George and St. Paul; Hatch, 1993). The remaining Alaska fulmar population is spread across a few dozen smaller colonies, each with 1s to 1000s of birds (USFWS, 2006). While numerous in Alaska waters, fulmars are challenging to study due to their highly pelagic distribution and the difficulty of accessing breeding colonies on remote island cliffs. Given the relatively large estimated population of fulmars, fisheries bycatch is assumed to be demographically insignificant. However, studies employing satellite telemetry (Hatch et al., 2010) and at‐sea surveys (Renner et al., 2013) suggest that fisheries may disproportionately affect certain breeding colonies.

Genetic stock identification (GSI) can provide estimates of bycatch percentages from sufficiently distinct populations. GSI has been applied to understand population‐level movement across geographic space in a variety of animals that are largely philopatric (e.g., Garza et al., 2014; Hasselman et al., 2016; Ruegg et al., 2014), a characteristic among seabirds that tagging studies suggest is also true for fulmar colonies in Alaska (Hatch et al., 2010). GSI requires reference data for a set of genetic markers from potential source populations to provide allele frequency estimates for these markers. When bycatch is genotyped with the same set of genetic markers, individual samples can be assigned to source populations using maximum likelihood or Bayesian inference (Pella & Masuda, 2000; Smouse et al., 1990). In high gene flow species, markers with greater differentiation than the genetic background may increase resolution for assignment (Nielsen et al., 2012) and without such markers, GSI may be of limited utility (Colston‐Nepali et al., 2020).

Here, we evaluate whether bycatch may differentially affect individual breeding colonies of fulmars in Alaska. We (1) developed a set of novel genetic markers, (2) genotyped samples collected at the four largest breeding colonies with these markers, (3) used GSI to assign fulmar bycatch to source colonies, and (4) assessed the difference between the breeding colony population size and the percentage of bycatch assigned to each colony. Finally, to illustrate potential differences in exposure to fisheries, we calculated the overlap between spatial distributions of bycatch from each colony and analyzed these spatial distributions in the context of fishing effort data. Our results indicate that targeting 100–150 divergent loci is sufficient for GSI, despite high gene flow among fulmar populations. GSI has great potential for seabird conservation by using information about fisheries‐caused mortality to inform research and management priorities.

## 2 METHODS

### 2.1 Colony samples and population structure

**FIGURE 1:**
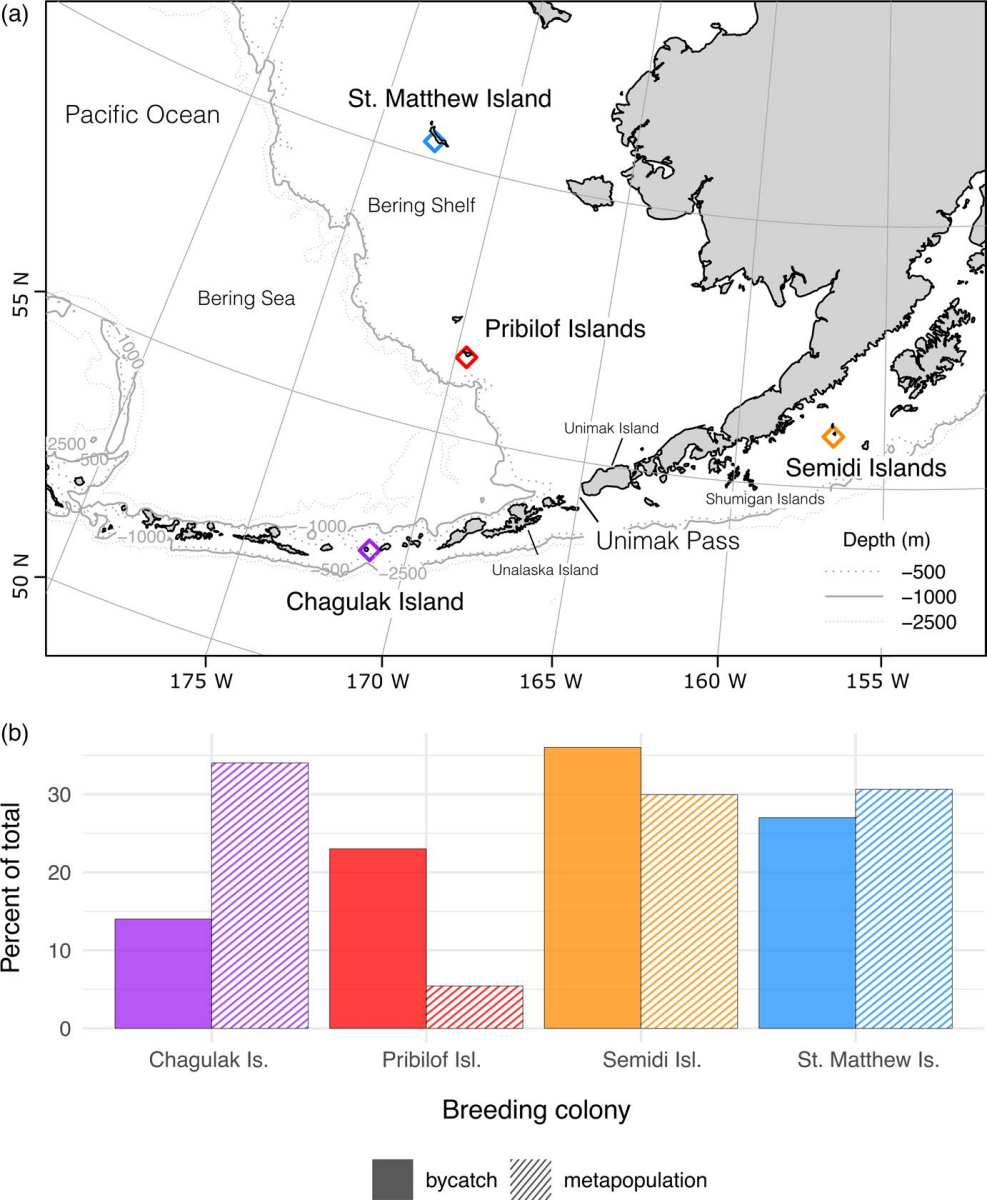
Geographic locations for the four major Pacific Northern Fulmar breeding colonies in Alaska (a) and the percent of the total metapopulation (population size) and total bycatch assigned to each colony (b). Most recent colony population estimates are as follows: Chagulak (~500,000); Semidi Islands (~440,000); St. Matthew Island (~450,000); and the Pribilof Islands (~79,700). Colony locations are indicated with open diamonds. Dominant oceanographic features are shown, including the Bering Shelf and Unimak Pass

Blood or tissue was collected from fulmars at the four largest Alaska breeding colonies (Chagulak Is., St. Matthew and Hall Isl., Pribilof Isl., and Semidi Isl., Figure 1A) from 1992 to 2004 under U.S. Geological Survey Bird Banding permit #20022.

To identify genetic markers for distinguishing fulmars from the four breeding colonies, we used restriction site‐associated DNA sequencing (RADseq) with 24 samples per colony as in Ruegg et al. (2018; Data S1). Samples were selected based on DNA concentration (> 10 ng/ µl) and the presence of high molecular weight bands in a 1% agarose gel, and then normalized to 150 ng in 15 µl using the EpMotion pipetting robot (Eppendorf) prior to library preparation. DNA was digested with the *SbfI* restriction enzyme (New England Biolabs, NEB) and a target fragment length of ~500 bp (see Data S1 for details of RADseq protocol).

Sequence data were analyzed following Ruegg et al. (2018) using filtering in *Stacks* (v1.48, Catchen, 2013; details in Data S1). Filtered reads were aligned to the fulmar genome (Zhang et al., 2014) with bowtie2 (v2.3.2, Langmead & Salzberg, 2012) and SNPs identified using the Haplotype Caller in the Genome Analysis Toolkit (GATK, v3.4). SNPs were quality filtered in VCFtools (v0.1.13, minimum genotype quality <30; minimum read depth <8; minor allele frequency <0.01; removing indels and nonbiallelic SNPs; Danecek et al., 2011). SNPs called in <20% of samples (20 of 96) were removed to reduce the number of low‐coverage SNPs and minimize the amount of missing data per genotype. Then, to further reduce the level of missing data per individual, we plotted the fraction of missing genotypes per individual and fraction of missing genotypes per locus to determine a threshold of individuals and SNPs to retain. The filtered dataset was then used to assess population structure among colonies and to design genetic markers for GSI.

We calculated F_ST_ using one SNP per genome scaffold or SNPs >100 kb apart on scaffolds longer than that distance to reduce the potential influence of linked SNPs (linkage disequilibrium; LD). Samples with >20% missing data were removed prior to analysis to avoid artifacts from missing data.

### 2.2 Genetic marker discovery

For designing GSI markers, we used the filtered RADseq dataset, which included 105,000 SNPs and 67 fulmars (29 samples did not pass the quality filtering and missing data criteria). To identify outlier SNPs from the RADseq data to differentiate breeding colonies, we calculated the per colony allele frequency at each of the variant sites, and then compared those frequencies among each pair of colonies to create a list of variants with the greatest allele frequency differences between each pair of colonies. We picked the top 40 of these divergent SNPs for each comparison and extracted 75 bp flanking the target SNP from the Northern Fulmar genome. When multiple SNPs occurred within <150 bp, we attempted to include these microhaplotype loci by designing primers that captured multiple variants. Microhaplotype markers can increase resolution for population genetic analyses, including genetic assignment (McKinney et al., 2017). Primers were selected using the software Primer3 (Rozen & Skaletsky, 2015) in Geneious (v10.2, Kearse et al., 2012). Amplicon loci targeted 100–120 bp including primer regions for compatibility with 2x 75 bp Illumina sequencing.

To complete primer design for sequencing library preparation using Genotyping‐in‐Thousands by sequencing (GT‐seq; Campbell et al., 2015), we added Illumina sequencing primers to the locus‐specific primers as described in Baetscher et al. (2018). We tested multiplex amplification and sequencing of 144 short DNA fragments (amplicon loci) with 192 colony samples and called genotypes using the workflow in Baetscher et al. (2018). In brief, this workflow combines paired‐end reads using the Fast Length Adjustment of Short reads (FLASH v1.2.11 Magoč & Salzberg, 2011), maps reads to a reference FASTA file using the Burrows–Wheeler Aligner (BWA‐MEM v0.7.16, Li & Durbin, 2009), and then converts the output Sequence Alignment Map (SAM) files to Binary Alignment Map (BAM) files with SAMtools (v1.5, Li and Durbin, 2009). BAM files were used as input for calling variants in FreeBayes (v1.1.0, Garrison & Marth, 2012) with flags to exclude multinucleotide polymorphisms (MNPs) and complex polymorphisms. The Variant Call Format (VCF) file was filtered to include variants with a minimum base quality of 30, depth of 10 reads, and no indels using VCFtools (Danecek et al., 2011; additional details in Data S1).

The filtered VCF file and aligned SAM files were used as input for the R Shiny app, microhaplot (v1.0.1, Ng & Anderson, 2019), a software program that generates genotype data from the phased haplotypes included in the individual SAM files in conjunction with the VCF file and allowed us to evaluate each locus based on the fraction of callable haplotypes, the number of haplotypes present, and HWE. Genotypes were filtered in R (R Core Team, 2020) for a minimum of 20 reads per individual/locus and those with an allelic ratio less than 0.4; both filters remove extraneous alleles generated by sequencing errors and index switching. Individuals with missing data at more than 28 loci (20% missing data) were removed and then the dataset was checked for matching genotypes, which would be indicative of duplicate samples.

HWE for each locus/population and expected and observed heterozygosities were calculated using hierfstat in R (Goudet, 2005), and loci that deviated from HWE in three or more populations were removed. To evaluate the statistical power of the new markers for population assignment, we used self‐assignment of samples to known source colonies with the Bayesian likelihood method in the R package rubias (Moran & Anderson, 2018; details in Data S1).

### 2.3 Fisheries bycatch samples

Fulmars were collected dead by NOAA observers (USFWS permit MB035470‐0) through the North Pacific Observer Program (Observer Program) from 2006 to 2017. The Observer Program monitors the groundfish fisheries, which occur in the Bering Sea, Aleutian Islands, and Gulf of Alaska, and target Pacific halibut (*Hippoglossus stenolepis*), Pacific cod (*Gadus macrocephalus*), walleye pollock (*Gadus chalcogrammus*), and sablefish (*Anoplopoma fimbria*; Eich et al., 2018). Observers collect up to five fulmars per cruise and observer coverage depends on vessel type, participation in the fishery, and limited access programs (NOAA, 2019). Birds collected for examination represent 4% of fulmars caught during our 11‐year sampling period. Fulmars were necropsied and 1 ml of pectoral muscle was sampled and frozen at −20°C.

### 2.4 Bycatch assignment

We genotyped bycatch using the genetic markers designed and validated in breeding colony samples and then assigned bycatch to colony of origin based on genotype data (details in Data S1). Because unequal sample sizes for reference populations can affect GSI assignment (Paetkau et al., 2004), we used downsampled genotype data from the two largest colonies in the reference dataset for bycatch GSI with rubias and retained samples assigned at 90% probability (details in Data S1 and code available in GitHub repository).

If bycatch were equally distributed among breeding colonies, we would expect the majority of bycatch to originate from the three largest colonies (Chagulak Is., St. Matthew and Hall Isl., and Semidi Isl.), with a smaller percentage of bycatch from the Pribilofs. To test this hypothesis, we compared bycatch GSI assignments to colony population size and used Pearson's Chi‐squared tests to determine whether observed bycatch percentages differed from the expected percentages based on colony size (Gotelli & Ellison, 2013). Tests used a Bonferroni sequential adjustment (Holm, 1979) to account for multiple comparisons.

### 2.5 Bycatch spatial distributions

To assess the extent to which at‐sea distributions of bycatch from each of the four colonies were distinct, we estimated utilization distributions (UDs) based on fishery observer locations for bycatch birds and quantified the degree of similarity between UDs from different colonies during the breeding (May–Sept.) or nonbreeding (Oct.–April) season with Bhattacharyya's Affinity (BA; Bhattacharyya, 1943). Core use areas are represented by the 50% UD and values range from 0 to 0.5 (no overlap to complete overlap; Fieberg & Kochanny, 2005). We compared the 50% UD for bycatch for each pair of colonies in the breeding and non‐breeding seasons (12 pairwise comparisons) and then ranked these comparisons from the least‐to‐most similar distributions.

### 2.6 Fishing effort

We obtained data for longline fisheries from the Alaska Fisheries Information Network (AKFIN; NOAA Alaska Fisheries Science Center, 2021) for the Bering Sea, Aleutian Islands, and Gulf of Alaska from 2006 to 2017. Longline gear accounted for 87% of seabird bycatch and 86% of fulmar bycatch from 2010 to 2017, with the remaining bycatch taken using different gear types, primarily trawl (Krieger et al., 2019). Here, we use only longline fishing effort data due to the fact that the majority of fulmar bycatch originates from this gear type. Vessel locations (latitude and longitude) in spatial grids were rounded to 0.5 degrees and then the number of hooks was aggregated within those grid cells to capture fishing effort for the bycatch sampling period.

## 3 RESULTS

### 3.1 Genetic marker discovery and population structure

Filtered RADseq data included 105,000 SNPs and genotypes for 67 fulmars (16–18 per colony; missing SNP data per bird = 0.4–81.4%, mean = 13.8%, see SI for details). Prior to using these data for estimates of population structure, we further filtered SNPs to minimize linkage disequilibrium and high missing data rates (>20%) and calculated pairwise F_ST_ for 15,917 SNPs and 52 samples, with values ranging from 0.003 to 0.01 (Table 1).

**TABLE 1:** Spatial analysis of overlap in 50% utilization distributions (UDs) between bycatch assigned to different breeding colonies using Bhattacharyya's Affinity (BA). With 50% UDs, complete overlap corresponds to a BA value of 0.5. The breeding season is May–September. Pairwise F_ST_ is calculated from 15,917 SNPs from RADseq data

Colony comparison	Season	BA (50% UD)	F_ST_ (pairwise)
St. Matthew	Breeding	0.255	0.0093
Chagulak			
St. Matthew	Nonbreeding	0.291	
Chagulak			
Pribilof	Breeding	0.304	0.011
Chagulak			
Pribilof	Nonbreeding	0.295	
Chagulak			
Pribilof	Breeding	0.324	0.0075
Semidi			
Pribilof	Nonbreeding	0.286	
Semidi			
Semidi	Breeding	0.331	0.0045
Chagulak			
Semidi	Nonbreeding	0.303	
Chagulak			
St. Matthew	Breeding	0.338	0.005
Semidi			
St. Matthew	Nonbreeding	0.323	
Semidi			
St. Matthew	Breeding	0.351	0.0033
Pribilof			
St. Matthew	Nonbreeding	0.386	
Pribilof			

We genotyped 611 colony samples with 141 of the original 144 loci (conversion rate = 98%; Table S1), and then removed 8 loci that deviated from Hardy–Weinberg (*p* < 0.05) in at least three populations. We also removed data for one copy each for 13 birds with duplicate genotypes and 81 samples with >20% missing data. Genetic data for the remaining 517 samples included 551 alleles (mean = 3.9 alleles/locus; range = 1–10 alleles/locus).

Using a 90% likelihood criterion for genetic self‐assignment, 56.5% of birds were assigned to a single breeding colony, with the remaining known colony birds assigned at lower levels of probability. Using that same 90% likelihood criterion, self‐assignment accurately identified 91.4% of samples to their colony of origin (range = 50% for St. Matthew to 95.9% for the Semidis; Table S3a). Among samples not assigned to the correct colony, the largest percentage of misassignments occurred in the colonies with the smallest sample sizes: Chagulak and St. Matthew (Table S4).

The two colonies with the largest sample sizes (Semidis and Pribilofs) had significantly higher self‐assignment accuracy (χ^2^; *p* = 0.017, df = 3). Equalizing the sample size for each colony to 36 improved assignment accuracy for Chagulak (75% to 83.3%) and St. Matthew (50% to 54.5%). However, assignment accuracy decreased for the Pribilofs (91.3% to 83.3%) and Semidis (95.9% to 60%), and overall accuracy decreased (Table S3b).

### 3.2 Bycatch assignment

Ninety‐eight percent of bycatch samples genotyped (1,507) passed quality filtering steps for sequencing read depth, allelic ratio, and missing data. GSI assigned 68% of bycatch to one of the four breeding colonies with a 90% likelihood criterion. The smallest percentage of bycatch was assigned to Chagulak (13.7%) and the largest to the Semidis (35.7%). St. Matthew and the Pribilofs comprised 27.3% and 23.3% of the bycatch, respectively (Table 2; Figure 1B).

**TABLE 2:** Summary of the percent of bycatch samples assigned to each colony compared to (a) the percent of the region's total population that each colony comprises (“across colonies”, *n* = 1027), and (b) the percent of bycatch from each season within a colony (“within colonies by season”, *n *= 1009)

Colony	Pop. Est.	% total pop.	*n* assigned	% bycatch	χ2(1)	*p*‐value	*n* with season data	Season	*n* by season	% bycatch (*n* with season data)	% bycatch within colony
Semidis	440,000	30	367	36	1.7143	0.3808	360	Breeding	166	16	46
								Non‐breeding	194	19	54
Pribilofs	79,700	6	239	23	52.2411	**<0.0001**	236	Breeding	117	12	50
								Non‐breeding	119	12	50
St. Matthew/Hall	450,000	30	280	27	0.4286	0.5127	274	Breeding	133	13	49
								Non‐breeding	141	14	51
Chagulak	500,000	34	141	14	17.8253	**<0.0001**	139	Breeding	72	7	54
								Non‐breeding	62	6	4

Note

We used >90% likelihood thresholds for bycatch assignments. Pearson's Chi‐square, degrees of freedom (in parentheses), and *p*‐values test the percentage of the total population for each colony against the percentage of bycatch originating from that colony. Bolded *p*‐values represent significance at *p* < 0.05. Tests used a Bonferroni sequential adjustment (Holm, 1979).

Bycatch assigned to the Pribilofs was disproportionately high relative to the percentage of fulmars estimated to breed there (23% of bycatch; 6% of the metapopulation; Table 2). Additionally, the percentage of bycatch inferred to originate from Chagulak was significantly lower than would be expected based on population proportions (14% of bycatch; 34% of the metapopulation; Table 2). Bycatch originating from the Semidis and St. Matthew did not differ significantly from expected percentages based on colony size (36% and 27% of bycatch, respectively; both colonies are ~30% of the overall metapopulation; Table 2).

### 3.3 Utilization distributions and spatial overlap among bycatch

**FIGURE 2:**
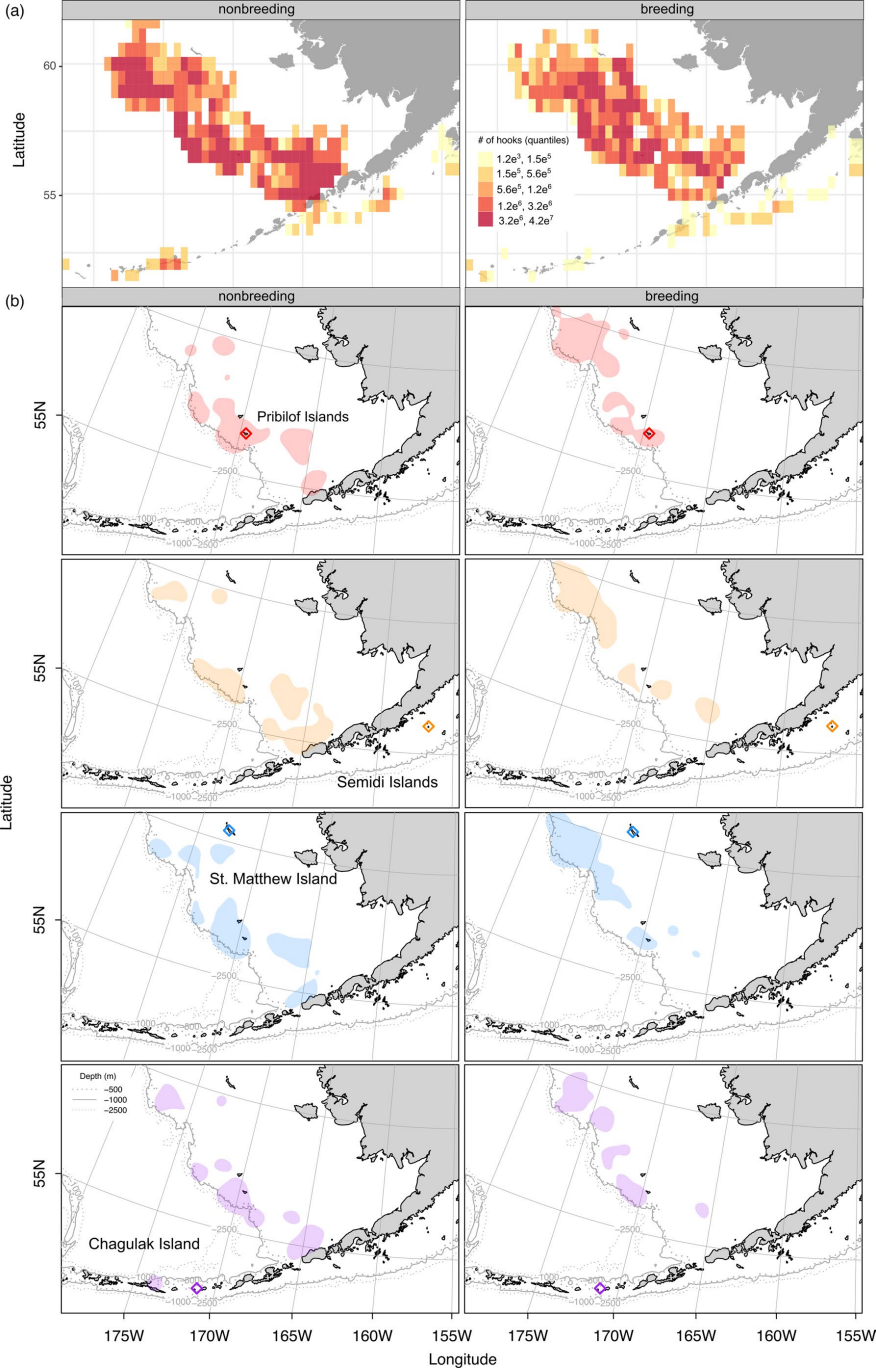
Fishing effort (aggregated number of hooks) for the longline fishery from 2006 to 2017 and core areas of Northern Fulmar bycatch for each of the four major Alaska breeding colonies. Fishing effort (a) and bycatch (b) are focused on the Bering Shelf for all colonies in both the nonbreeding (winter) and breeding (summer) seasons. Polygons depict 50% utilization distributions (UD). Colony locations are indicated with open diamonds and colony colors correspond to polygon color. Distributions of fulmars outside of fishery bycatch samples are not shown

Spatial analyses relied on bycatch assigned using GSI (i.e., distributions of birds that were not obtained as bycatch are not part of this dataset). Core spatial distributions for bycatch from each pair of colonies overlapped during both breeding and nonbreeding seasons. The Observer Program provided spatial and temporal data for 1,002 of the 1,027 bycatch samples that were assigned at >90% likelihood to a single colony. Spatial distributions of bycatch from different colonies were more similar during the nonbreeding season than during the breeding season (nonbreeding BA values: 0.286–0.386; breeding BA values: 0.255–0.351; Table 1). Bycatch from Chagulak and St. Matthew showed the least amount of overlap during the breeding season (50% UD BA = 0.255; Figure 2), followed by Chagulak and the Pribilofs (BA = 0.304), with the most overlap between St. Matthew and the Pribilofs (0.351; Figure 2). In the nonbreeding season, bycatch from St. Matthew and the Pribilofs had the greatest spatial overlap (BA = 0.386), while the Pribilofs and Semidis showed the smallest overlap (BA = 0.286).

### 3.4 Fishing effort

The aggregated number of longline hooks per 0.5‐degree grid cell over the sampling period ranged from 1,184 to 10.8 million during the breeding season (summer) and 4,370 to 41.7 million during the nonbreeding season (fall, winter; Figure 2A). The nonbreeding season consisted of nearly twice as many hooks in total (771 million) compared to the breeding season (396 million); yet the nonbreeding season (October–April) makes up 7 months, indicating that fishing effort is 1.4 times higher during the nonbreeding season. Areas of intense fishing effort along the Bering Shelf overlap with the core bycatch distributions for one or more of the breeding colonies (Figure 2; Figure S2), suggesting that, generally, more bycatch occurs in areas with greater fishing effort.

## 4 DISCUSSION

Tracking anthropogenic impacts to wildlife requires data on patterns of mortality and overlap between species and conservation threats. Here, we used GSI to identify the relative impact of fisheries bycatch among four populations within a larger metapopulation. We found disproportionately high levels of fulmar bycatch from the Pribilofs, the smallest breeding colony in Alaska, and disproportionately low levels from the Chagulak breeding colony, the largest in Alaska. As one of the most frequently caught seabird species in fisheries, fulmars can serve as a model for demonstrating the applicability of GSI to seabird bycatch, in particular for species or populations that are of high conservation concern.

### 4.1 Oceanography drives fishing effort and fulmar distributions

Overlap of fisheries with seabirds from different colonies can depend on the birds’ foraging ecology, but also the proximity and density of fishing effort if birds rely on fisheries discards as a food source (e.g., Soriano‐Redondo et al., 2016). In Alaska, fulmars are widespread throughout the eastern Bering Sea and Aleutian Islands. In the summer, at‐sea surveys show higher densities of fulmars along the Bering Shelf, near Unimak Pass, and around the four largest colonies, consistent with evidence that distance from colony is the strongest predictor of at‐sea density during the breeding season (Renner et al., 2013). That same study found fulmar distributions were driven by oceanographic variables (sea surface temperature, location, and primary production) and fisheries locations, although fishing effort may track some of these same oceanographic variables as they relate to the distribution of target commercial species. For longline fisheries, the Bering Shelf and, to a lesser degree, the Aleutian Island chain are high‐productivity regions that receive substantial fishing effort (Thompson, 2018; Figure 2A). Dietary evidence suggests that fulmars rely on fisheries offal and discarded bait as a key food source (Phillips et al., 1999) and that birds are directly attracted to vessels fishing in highly productive waters. However, the extent of reliance on discards is unclear, as multiple studies have indicated that bird distributions are more closely correlated with oceanographic variables than with fishery discards (Renner et al., 2013) or that diets at certain colonies are dominated by natural prey (e.g., Furness & Todd, 1984). Camphuysen and Garthe (1997) further suggest that fulmars can be locally attracted to vessels as they forage naturally rather than following vessels, or that reproductive energetics and competition with other avian species influence the extent to which fulmars rely on discards. Despite some ambiguity about the mechanisms underpinning interactions between fulmars and groundfish fleets, both birds and fishing fleets have shifted northward in the Bering Sea as the water warms and fish stocks move north (Renner et al., 2013).

Bycatch samples in our study primarily reflect interactions between fulmars and the Bering Sea Pacific cod longline fishery, where >85% of bycatch originates (Krieger et al., 2019). Vessels in this fishery harvest Pacific cod throughout the Bering Sea and Aleutian Islands (Thompson, 2018; Figure 2A). Pacific cod are abundant and widely distributed along the Bering Shelf in the summer (and to a lesser extent along the Aleutian Islands), with a fall migration seaward to shelf breaks. Dense aggregations in the winter occur around spawning grounds, often on the Bering Sea side of Unimak and Unalaska Islands and southwest of the Pribilof Islands, as well as near the Shumagin Islands in the western Gulf of Alaska (Shimada & Kimura, 1994). Within the North Pacific groundfish fisheries management regions, seasons for specific stocks and gear types open and close throughout the year such that fishing occurs year‐round, with fulmars present at many of the vessels. Fulmar bycatch occurs in the longline Pacific cod fisheries throughout the year, but increases in October–December (Melvin et al., 2015) after the Bering Sea walleye pollock trawl fisheries close for the season (Ianelli et al., 2020). Longline Pacific cod fisheries become the dominant source of fisheries discards in the region after the walleye pollock fishery has closed.

### 4.2 Central‐place foraging dictates seasonal colony‐specific bycatch

During the breeding period, spatial overlap (UDs) of bycatch from different colonies highlights the role of central‐place foraging in fulmar ecology. For example, bycatch from Chagulak, the colony which experienced the smallest amount of bycatch, shares the least spatial overlap with bycatch from St. Matthew (Table 1; Figure 2), likely because of their relative locations as the farthest north (St. Matthew) and farthest southwest (Chagulak) colonies, nearly 900 km apart (Figure 1). Central‐place foraging would separate breeding fulmars and may account for the overall lower bycatch of Chagulak birds if they are foraging farther from the Bering Shelf fisheries (Renner et al., 2013).

In contrast, the two colonies on the Bering Shelf, St. Matthew and the Pribilofs, had similar percentages of bycatch (27.3% and 23.3%, respectively; Table 2), and the greatest amount of spatial overlap for those bycatch samples (BA = 0.351, breeding, and 0.386, nonbreeding; 0.5 would be 100% overlap at 50% UD). This overlap coincides with shared exposure to year‐round fishing along the Bering Shelf.

Disproportionately high bycatch from the Pribilofs likely arises from a combination of continuous proximate exposure to fishing vessels and its relatively small colony size (Table 2). St. Matthew is home to nearly an order of magnitude more fulmars (30% of total fulmar population), and thus its percentage of bycatch (27%) is roughly equivalent to its population size. Similarly, the Semidi Islands sustained the largest percentage of bycatch (35.7%), yet comprise 30% of the total population, and Chagulak, with the largest estimated population (34% of total) accounted for just 13.7% of bycatch, presumably due to its location along the Aleutian Islands where there is less concerted fishing effort than the Bering Shelf (Figure 2A).

Birds from all colonies had spatial overlap in the winter with the Pacific cod longline fleet in areas of known dense spawning aggregations, especially southeast of the Pribilof Islands and around Unimak Pass. Although this pattern might suggest that as fulmars are released from central‐place foraging in the nonbreeding season (fall and winter), birds from all colonies may experience more similar risk of bycatch, our data show bycatch proportions remain fairly stable across the breeding and non‐breeding seasons (Table S2). A dataset of fulmar distributions independent of fishing effort (i.e., not derived from fisheries bycatch) might better capture seasonal changes (see Hunt et al., 2014; Suryan et al., 2016 for examples).

Notably, our inference of fulmar distributions relies on birds taken as bycatch. Distributions shown in Figure 2B do not include fulmars outside of bycatch samples, making it necessary to surmise that this missing component could contribute to lower bycatch rates if birds are preferentially foraging in areas distinct from the primary fishing effort (Figure 2A; Figure S2). Another consideration is that we used only longline fisheries data to quantify fishing effort in the present study, and despite longline fisheries comprising >85% of seabird bycatch, alternative gear types may expand the spatial footprint or intensity of fishing effort in the region.

### 4.3 Low genetic differentiation and demographic implications

Low genetic differentiation among fulmar breeding colonies (F_ST_ = 0.003–0.01) indicates substantial gene flow, either currently or in the recent past (Friesen, 2015), or that populations are recently separated and have not yet reached migration–drift equilibrium. These low F_ST_ values are consistent with a recent study of Atlantic fulmars (Colston‐Nepali et al., 2020); however, the demography of the Atlantic and Pacific subspecies is quite different: breeding fulmars in the Atlantic are distributed among many small colonies, whereas Pacific fulmars are concentrated at just a few large breeding colonies. Due to the remote nature of the Alaska colonies, there is little available evidence for demographic mechanisms that could contribute to low levels of genetic differentiation. However, postnatal dispersal of immature individuals to new colonies could be a contributing factor. Banding studies in the United Kingdom recorded 31% of banded fulmar fledglings returned to breed within 20 km of their natal colony (Coulson, 2016; Wernham et al., 2002), although it is unclear how much dispersal versus mortality contribute to the low return rate. For Alaska fulmars, high gene flow among colonies could buffer the impact of bycatch and mitigate a loss of genetic diversity; however, if colonies are more demographically independent than suggested by the available data, bycatch could present a greater threat to the Pribilof colony, particularly since disproportionate bycatch has likely persisted for decades given the longevity of the region's fisheries. Furthermore, bycatch could eventually influence effective population sizes due to the disproportionate removal of males, as has been found in fulmar bycatch from these same samples (Beck et al., 2021). Socially monogamous behavior in fulmars means that disproportionate removal of a single sex – which can leave a breeding bird without its mate – impacts the ability to raise chicks, thereby further reducing the breeding population.

Our results show spatial and temporal patterns of fisheries‐caused mortality at sea for individual breeding colonies and highlight a need for current colony size estimates to assess demographic trends, particularly at the Pribilofs. Management directives to use seabird deterrents have successfully decreased bycatch (Melvin et al., 2019), indicating that effective mitigation can help achieve conservation goals. In addition to deterrents, temporal closures have been implemented for similar purposes; for example, fisheries managers have seasonally limited harvest, and therefore bycatch, near colonies of conservation concern to protect Southern Ocean seabird species (Waugh et al., 2008).

GSI has broad utility for connecting anthropogenic mortality (e.g., bycatch, oil spills, and offshore wind projects) to discrete breeding populations of marine vertebrates. In our study, we show disproportionate take of fulmars from specific colonies in some of the largest fisheries in the United States and provide an example of how GSI can be applied to species with genetically structured populations, some of which are critically endangered. Global longline fisheries accidentally catch a variety of seabirds – notably, albatrosses (e.g., Jiménez et al., 2014; Melvin et al., 2019) and penguins (Crawford et al., 2017) – as well as sea turtles, elasmobranchs, and marine mammals (Jaiteh et al., 2021; Thorne et al., 2019). In many of these cases, characterizing population‐level impacts of bycatch require that animals are tracked or that a significant portion of the population is banded. However, GSI of bycatch samples obviates this requirement and can be the only feasible option when tracking and banding are not logistically possible (which is the case for many populations).

Advances in genetic sequencing technology and analyses, including the targeted microhaplotype loci used in this study, allow for GSI even when species exhibit very low levels of genetic differentiation. Although the additional discriminatory power of microhaplotypes compared to SNPs depends on the study species and allele frequency differences among populations, microhaplotypes are generally a more efficient marker type for generating equivalent (or greater) assignment power, particularly for species with large population sizes (and therefore, a tendency toward more SNPs within a single sequencing read; a comparison of the power of SNPs vs. microhaplotypes can be found in Baetscher et al., 2018). Furthermore, the software used for these GSI analyses includes information about whether samples are highly genetically dissimilar from reference populations, thus providing insight about bycatch from unsampled populations through the use of z‐scores (although this was not an issue in the present study, where 99% of Alaska fulmars breed on one of the four sampled colonies). Combining these genetic approaches with spatial and temporal interaction data can provide a clearer picture of areas of high risk and which stocks are susceptible to those risks, which can then inform more targeted and effective management actions. For species that face threats in multiple habitats, GSI is a critical tool for disentangling the impacts of such threats – both genetically and demographically – and can contribute to a more holistic understanding of the impacts of fisheries bycatch.

## CONFLICT OF INTEREST

The authors declare no conflict of interest.

## Supporting information

Supplementary Material

## Data Availability

The data for this study are available from DataONE, https://doi.org/10.24431/rw1k57v. Analyses are available on GitHub at https://doi.org/10.5281/zenodo.5998907.
