# Supplementary material for: Genetic assignment of fisheries bycatch reveals disproportionate mortality among Alaska Northern Fulmar breeding colonies
Source: Evol Appl. 2022 Mar 4;15(3):447–58. doi: 10.1111/eva.13357 (PMC8965376; doi:10.1111/eva.13357)
Supplement: Supplementary file 1 — Supplementary Material [file EVA-15-447-s001.docx]

**Supporting Information**

Genetic assignment of fisheries bycatch reveals disproportionate mortality among Alaska Northern Fulmar breeding colonies

**Laboratory protocol**

*Restriction-site Associated DNA sequencing*

Samples were selected based on DNA concentration (> 10 ng/µl) and the presence of high molecular weight bands in a 1% agarose gel. To ensure high-quality input DNA, we quantified 192 samples (48 from each breeding colony) with the broad range double-stranded DNA assay on a Qubit Fluorometer (Invitrogen) and then ran 2 µl of DNA extract diluted into 2 µl deionized water on a 1% agarose gel (135 W; 70 min) to ensure samples were uniformly composed of high molecular weight genomic DNA. We excluded low concentration (< 10 ng/µl) samples and those that smeared in the gel image, and then chose the 24 highest concentration samples from each colony for Restriction site-Associated DNA sequencing (RADseq; Baird et al. 2008). Samples were normalized to 150 ng in 15 µl using the EpMotion pipetting robot (Eppendorf) prior to library preparation.

DNA was digested with the SbfI restriction enzyme (New England Biolabs, NEB), which cuts DNA at specific sites that allow for ligating adapters. After ligation, samples were cleaned up with 1X Agencourt AMPure XP beads (Beckman Coulter), and then sheared using sonication on a BioRupter NGS (Diagenode) to an average length of 500 bp (10 cycles of 15 seconds on high power and 90 seconds off). Biotinylated ends on the adapters bind to Dynabeads M-280 streptavidin magnetic beads (Life Technologies), which ensure that only fragments with adapters are retained. DNA is liberated from the Dynabeads and cleaned up with AMPure XP beads before blunt fragment ends were repaired with the Illumina NEBNext Ultra DNA Library Prep Kit (New England Biolabs, NEB). NEBNext Adapters were then ligated onto the ends of the blunt DNA fragments. Again, AMPure XP beads were used to select ~500 bp fragments.

Prior to PCR enrichment of the sequencing library, we performed a test PCR with 5 µl DNA and 15 cycles to determine the appropriate number of cycles based on gel image and band brightness. Following the test PCR, we performed the final library amplification using 15 µl DNA. The PCR product was cleaned-up with AMPure beads and size distribution checked on a BioAnalyzer DNA High Sensitivity chip (Agilent) to ensure that minimal contaminants remained.

**Bioinformatics and data analysis**

Sequence reads were demultiplexed using sample barcodes, and subsequent filtering was performed in *Stacks* (v1.48, Catchen, 2013). Sequencing adapters and PCR duplicate read pairs were removed with the *process_radtags* and *clone_filter* functions, respectively, and only when both reads in a pair passed quality filters were they retained for further analyses. Filtered reads were mapped to the existing fulmar genome assembly (Zhang et al., 2014) with bowtie2 (v2.3.2, Langmead and Salzberg, 2012) and then SNPs were identified using the Haplotype Caller in the Genome Analysis Toolkit (GATK, v3.4) following GATK Best Practices (software.broadinstitute.org/gatk/best-practices). SNPs were filtered by removing indels, SNPs with a minimum genotype quality < 30; minimum read depth < 8; minor allele frequency < 0.01; and non-biallelic SNPs using VCFtools (v0.1.13, Danecek et al., 2011). Additionally, SNPs that were called in < 20% of samples (20 out of 96) were also removed with the goal of reducing the number of low coverage SNPs and simultaneously minimizing the amount of missing data per genotype.

Additional filtering steps were taken to reduce the amount of missing data per individual by plotting the fraction of missing genotypes in each sample by the number of individuals and also plotting the fraction of missing genotypes per locus. From these plots, thresholds for the number of SNPs and individuals to retain were chosen. Because our primary goal was to use these data to identify markers for GSI, rather than using the RADseq data for genotyping-by-sequencing, a higher rate of missing data was tolerated.

*Microhaplotype genotyping*

Sequencing libraries were prepared in plates of 96 samples using the GT-seq protocol: the first sequencing run included 192 samples and 144 microhaplotype loci to test multiplex amplification and the distribution of reads across loci, and the second contained 384 samples and 141 loci. Both libraries were genotyped on a MiSeq (Illumina) using paired-end 75 bp sequencing.

Initial assignment of sequencing reads to individual samples was performed using the MiSeq Analysis Software, and genotypes were obtained using a bioinformatics workflow similar to Baetscher et al. (2018). Briefly, paired-end reads were combined with a minimum overlap of 10 bp and maximum overlap of 100 bp using the Fast Length Adjustment of SHort reads (FLASH v1.2.11, Magoč and Salzberg, 2011). Combined reads were mapped to a reference FASTA file using bwa mem (v0.7.16, Li and Durbin, 2009), and then Sequence Alignment Map (SAM) files were converted to Binary Alignment Map (BAM) files with SAMtools (v1.5, Li et al., 2009). BAM files were used as input for calling variants in FreeBayes (v1.1.0, Garrison and Marth, 2012). Samples with fewer than 10 reads at more than 10 loci were excluded to avoid artifacts caused by poor DNA quality. All remaining samples were included in the FreeBayes analysis, which output a variant call format (VCF) file excluding multi-nucleotide polymorphisms (MNPs) and complex polymorphisms. Additionally, the VCF was filtered to include variants with a minimum base quality of 30, depth of 10 reads, and no indels using VCFtools (Danecek et al., 2011).

*Breeding colony genetic assignment*

Self-assignment used the Bayesian likelihood method with the R package rubias (Moran and Anderson, 2018). After performing the self-assignment, we removed samples that were used for microhaplotype design from assignment results to avoid inflating accuracy estimates (Anderson, 2010). Accuracy is generally higher for populations with larger sample sizes, and individuals from populations with fewer samples can be mistakenly assigned to populations with larger sample sizes (Paetkau et al., 2004). To test whether sample size had an effect on self-assignment results among fulmar colonies, we randomly selected genotypes for 36 birds (the number of genotypes for the colony with the fewest samples) from the three colonies with larger sample sizes and re-ran the self-assignment analysis using an equal number of samples per colony (code at https://github.com/dbaetscher/fulmar-bycatch-GSI).

*Bycatch genetic stock identification (GSI)*

We assigned bycatch to colony-of-origin using breeding colony genotype data as the reference for assignment. Because sample size differences can bias assignments toward groups with larger reference sizes (Paetkau et al., 2004), we downsampled genotype data for the two largest colonies and used these downsampled data as the reference. We performed GSI of bycatch samples with the *infer_mixture* function in RUBIAS (Moran and Anderson, 2018) applying the MCMC method with 10,000 repetitions, and discarding the initial 1000 repetitions as burn-in. We retained data for samples assigned to a breeding colony at a 90% probability-of-assignment and discarded assignments below this threshold. To ensure that downsampled data were representative of the full dataset, we performed 100 repetitions of the downsampling procedure and genetic assignment and then retained data for samples that were assigned to the same colony (at the 90% likelihood threshold) in > 90% of downsampling repetitions (code available at https://github.com/dbaetscher/fulmar-bycatch-GSI). This approach removed infrequent (< 10% of iterations), inconsistent, and low probability assignments.

*Utilization distributions and spatial overlap among bycatch*

Fishery interaction data for bycatch interactions are confidential under the Magnuson-Stevens Act (reauthorized in 2019). Code for UDs and spatial overlap analyses is available at <https://github.com/dbaetscher/fulmar-bycatch-GSI>.

**SI Tables**

Table S1. Samples from the major Alaska Northern Fulmar breeding colonies included in Restriction-site-associated-sequencing (RADseq) and microhaplotype genotyping (mhaps).

| **Colony** | **Samples**  **Genotyped (RADseq)** | **Samples filtered (RADseq)** | **Samples genotyped (mhaps)** | **Genotypes filtered (mhaps)** |
| --- | --- | --- | --- | --- |
| Chagulak | 24 | 17 | 68 | 36 |
| Pribilof | 24 | 16 | 183 | 169 |
| Semidi | 24 | 16 | 300 | 254 |
| St. Matthew | 24 | 18 | 60 | 58 |
| **Total** | 96 | 67 | 611 | 517 |

Table S2. Summary of bycatch samples assigned to each colony during the breeding and non-breeding seasons compared to the percent of the total population that each colony comprises. Bycatch assignments used >90% likelihood thresholds. Pearson’s χ^2^, degrees of freedom (in parentheses), and *p*-values test the percentage of the total population for each colony against the percentage of bycatch originating from that colony during each season. Bolded *p*-values represent significance at *p* < 0.05. Tests use a Bonferroni sequential adjustment (Holm 1979).

| **Colony** | **% total pop.** | ***n* with season data** | **season** | ***n* by season** | **% of bycatch within season** | **χ2(1)** | **p-value** |
| --- | --- | --- | --- | --- | --- | --- | --- |
| Semidis | 30 | 360 | breeding | 166 | 34 | 0.7619 | 0.3827 |
|  |  |  | non-breeding | 194 | 38 | 3.0476 | 0.2427 |
| Pribilofs | 6 | 236 | breeding | 117 | 24 | 57.4468 | **<.0001** |
|  |  |  | non-breeding | 119 | 23 | 51.2411 | **<.0001** |
| St. Matthew/Hall | 30 | 274 | breeding | 133 | 27 | 0.4286 | 1.0254 |
|  |  |  | non-breeding | 141 | 27 | 0.4286 | 1.5381 |
| Chagulak | 34 | 139 | breeding | 72 | 15 | 16.0873 | **<.0001** |
|  |  |  | non-breeding | 62 | 12 | 21.5686 | **<.0001** |

Table S3. (a) Genetic assignment results for all samples from each breeding colony and (b) for 36 samples from each breeding colony (with large colonies downsampled to the same sample size as the smallest colony represented).


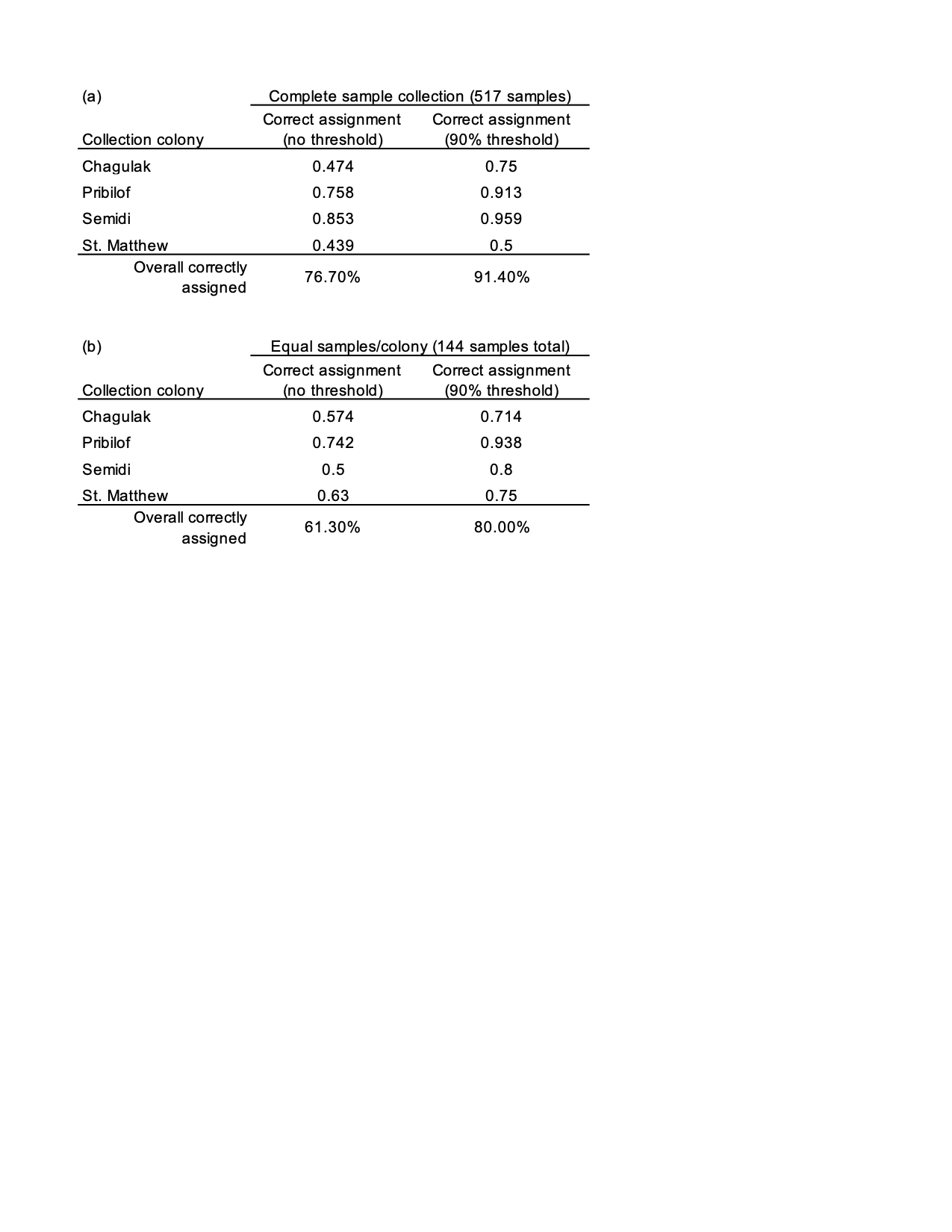


Table S4. Self-assignment results for all samples collected at breeding colonies with (a) no likelihood threshold and (b) a 90% likelihood threshold. Ascertainment samples were removed prior to assessing accuracy and are not included in the tallies. Gray shaded values indicate the number of accurate assignments.


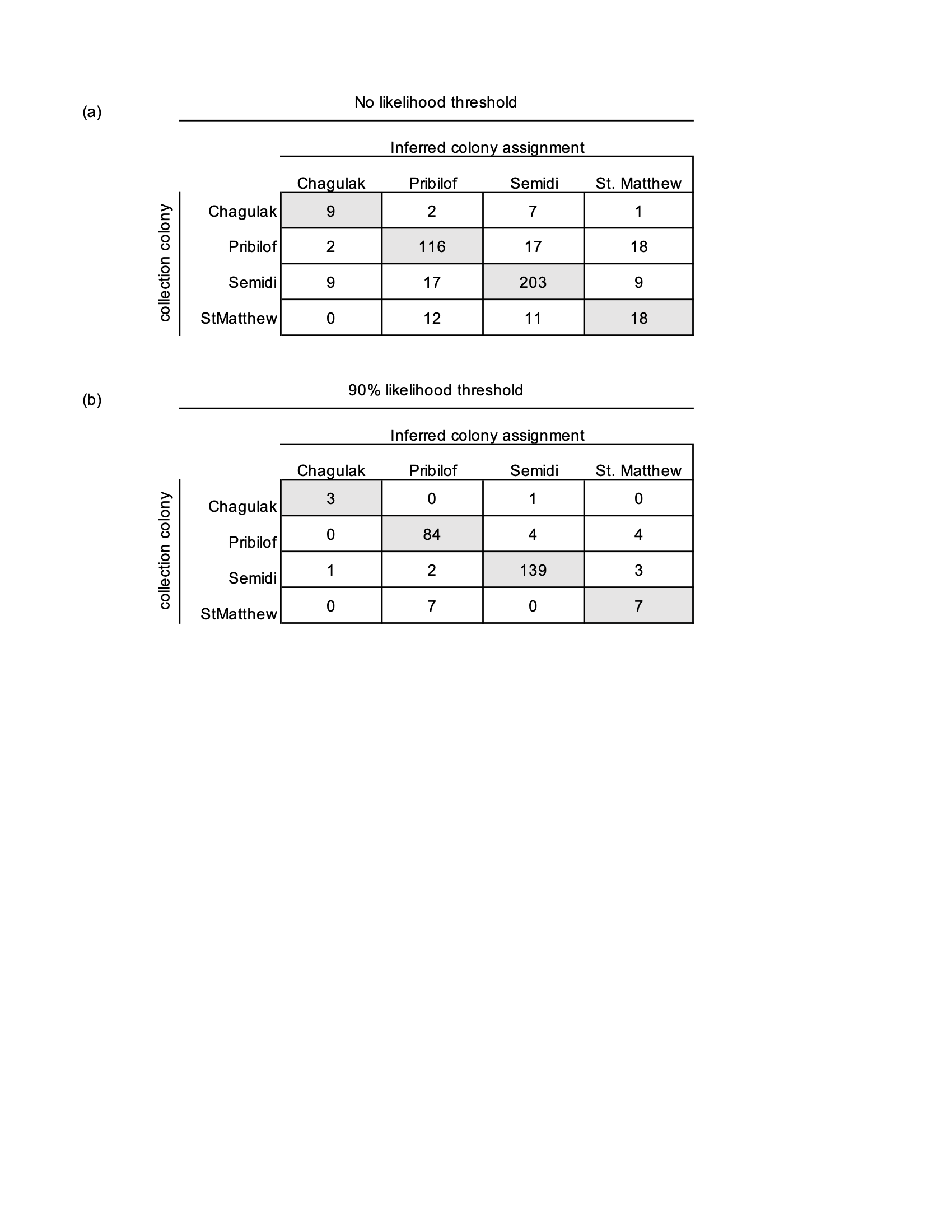


**SI Figures**

**
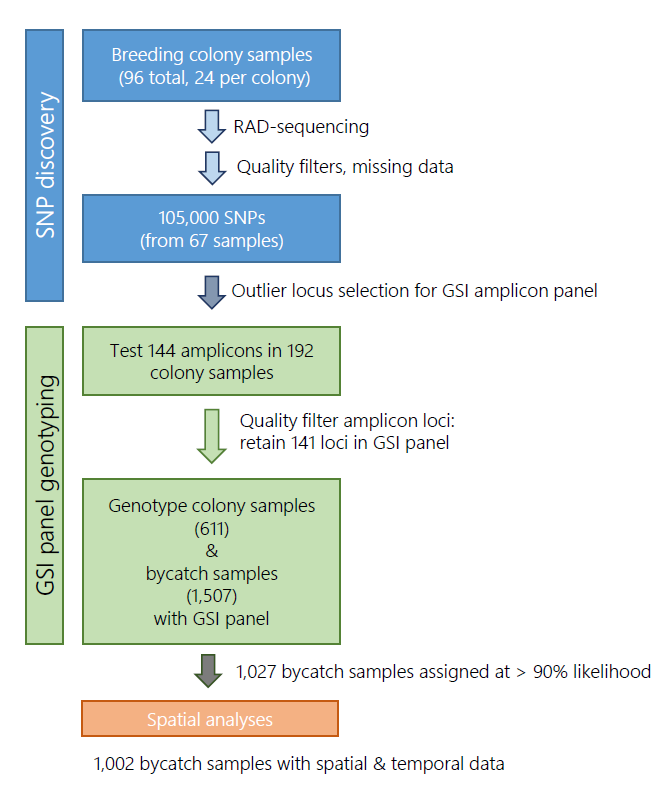
**

Figure S1. Experimental schematic of SNP discovery, locus-selection for the GSI amplicon panel, genotyping of colony and bycatch samples, and spatial analyses.


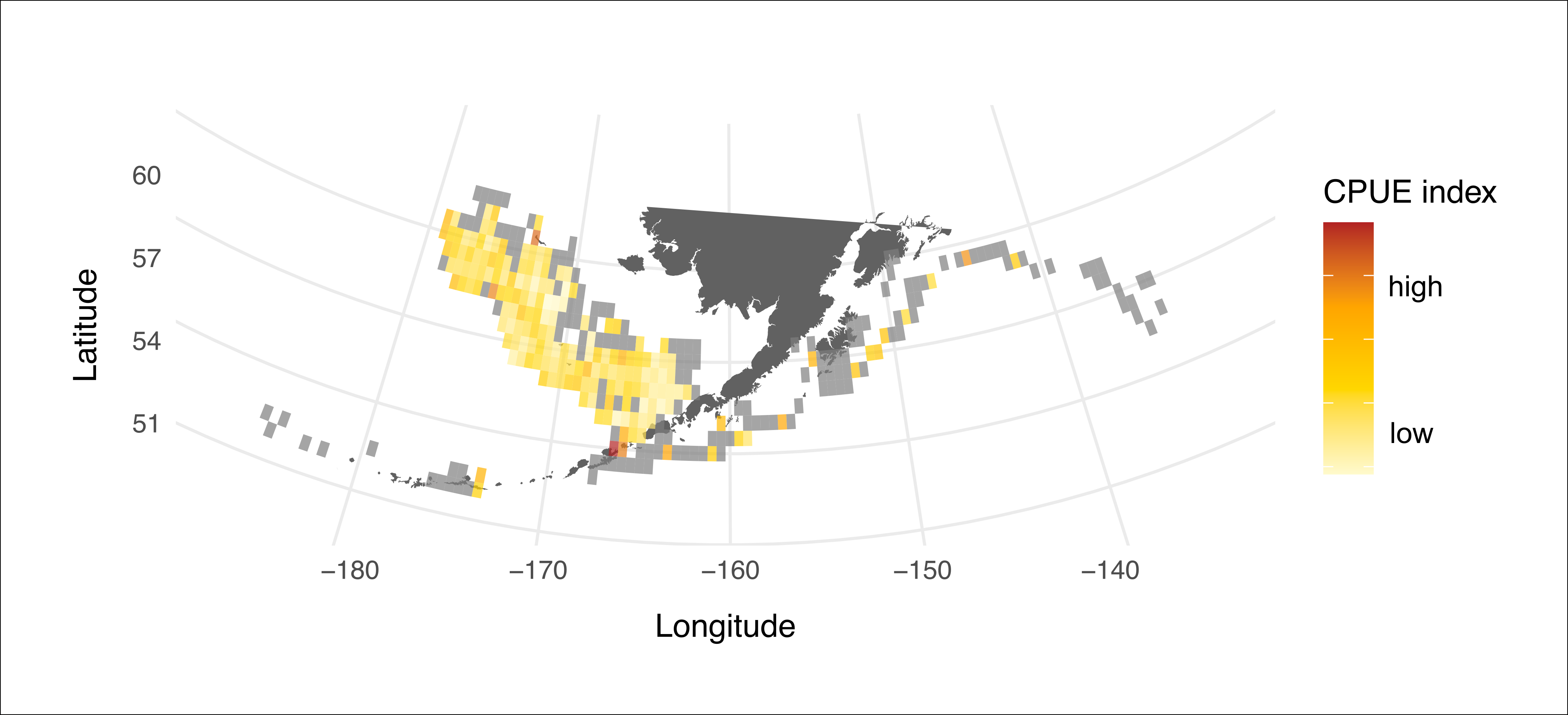


Figure S2. Catch per unit effort (CPUE) for Northern Fulmar bycatch based on longline fishing effort (number of hooks). Fisheries effort data is compiled from 2006-2017 to match the time period over which fulmar bycatch was obtained. CPUE has been transformed to a relative index of catch because fulmar bycatch data available in this study represents only 2% of fulmars caught in fisheries during this time period, while the effort data represents ~100% of longline effort. The map projection is Albers Equal Area.

**SI References**

Baird, N. A., Etter, P. D., Atwood, T. S., Currey, M. C., Shiver, A. L., Lewis, Z. A., … Johnson, E. A. (2008). Rapid SNP discovery and genetic mapping using sequenced RAD markers. *PLoS ONE*, 10. doi:10.1371/journal.pone.0003376.

Catchen, J.M. (2013). Stacks: An analysis tool set for population genomics. *Molecular Ecology*, 22, 3124–3140. doi: 10.1111/mec.12354.Stacks.

Danecek, P., Auton, A., Abecasis, G., Albers, C. A., Banks, E., DePristo, M. A., … 1000 Genomes Project Analysis Group (2011). The variant call format and VCFtools. *Bioinformatics*, 27, 2156–2158. doi: 10.1093/bioinformatics/btr330.

Garrison, E., Marth, G. (2012). Haplotype-based variant detection from short-read sequencing. arXiv:12073907v2 9. doi: arXiv:1207.3907

Goudet, J., Jombart, T. (2015). hierfstat: estimation and tests of hierarchical F-statistics

Holm, S. (1979). A simple sequentially rejective multiple test procedure. Scandinavian journal of statistics, 65-70.

Kearse, M., Moir, R., Wilson, A., et al (2012) Geneious Basic: An integrated and extendable desktop software platform for the organization and analysis of sequence data. Bioinformatics 28:1647–1649. doi: 10.1093/bioinformatics/bts199

Langmead, B., Salzberg, S. L. (2012). Fast gapped-read alignment with Bowtie 2. *Nature Methods,* 9, 357-359.

Li, H., Durbin, R. (2009). Fast and accurate short read alignment with Burrows-Wheeler transform. *Bioinformatics*, 25, 1754–1760. doi: 10.1093/bioinformatics/btp324.

Magoč, T., Salzberg, S.L. (2011). FLASH: Fast length adjustment of short reads to improve genome assemblies. *Bioinformatics,* 27, 2957–2963. doi: 10.1093/bioinformatics/btr507.

Ng, T.C., Anderson, E.C. (2016). MICROHAPLOT. doi: https://doi.org/10.5281/zenodo.820110.

Rozen, S., Skaletsky, H. (2015). Primer3 on the WWW for General Users and for Biologist Programmers. *Methods Molecular Biology*, 365–386. doi: 10.1385/1-59259-192-2:365.
